# Genome-wide association studies to assess genetic factors controlling cucumber resistance to CABYV and CMV in crop fields and the attractiveness for their *Aphis gossypii* vector

**DOI:** 10.1093/hr/uhaf016

**Published:** 2025-01-14

**Authors:** Séverine Monnot, Anaïs Ravineau, Eva Coindre, Pascale Mistral, Karine Leyre, Joel Chadœuf, Melissa Cantet, Nathalie Boissot

**Affiliations:** INRAE, Génétique et Amélioration des Fruits et Légumes, allée des chênes, 84143 Montfavet, France; Bayer Crop Science, Vegetable Trait Discovery, Mas Lamy, 13670 Saint-Andiol, France; INRAE, Génétique et Amélioration des Fruits et Légumes, allée des chênes, 84143 Montfavet, France; INRAE, Génétique et Amélioration des Fruits et Légumes, allée des chênes, 84143 Montfavet, France; INRAE, Génétique et Amélioration des Fruits et Légumes, allée des chênes, 84143 Montfavet, France; INRAE, Génétique et Amélioration des Fruits et Légumes, allée des chênes, 84143 Montfavet, France; INRAE, Génétique et Amélioration des Fruits et Légumes, allée des chênes, 84143 Montfavet, France; Bayer Crop Science, Vegetable Trait Discovery, Mas Lamy, 13670 Saint-Andiol, France; INRAE, Génétique et Amélioration des Fruits et Légumes, allée des chênes, 84143 Montfavet, France

## Abstract

Cucumber crops face high pressure from pathogens, including various viral species. Mapping quantitative trait loci (QTL) for vegetable resistance to viruses has primarily been conducted after mechanical inoculation in controlled environments, but not in crop field conditions. Moreover, viruses that cannot be mechanically inoculated, e.g. the cucurbit aphid-borne yellows virus (CABYV), have been overlooked in resistance studies. Here, we aimed to identify QTLs reducing epidemics of two prevalent cucumber viruses: CABYV and the cucumber mosaic virus (CMV). We evaluated the resistance of 256 elite cucumber lines and landraces in crop field conditions by screening for the presence of both viruses six-times during the season. We mapped twelve QTLs reducing CABYV epidemics and seven QTLs reducing CMV epidemics by combining multiloci genome-wide association studies and local score approach analyses. We also examined the attractiveness of this cucumber panel for *Aphis gossypii*, a major cucumber virus vector. We identified five QTLs that reduced the attractiveness, including one co-localizing with a QTL reducing CABYV epidemics. Interestingly, some accessions deemed CMV-resistant after mechanical inoculation in controlled environments showed high infection rates in crop field conditions. Only one QTL for CMV resistance was detected in both conditions, indicating that these phenotypes are regulated by independent QTLs. Local linkage disequilibrium study findings suggested that certain QTLs reducing epidemics were introduced early into elite lines through serendipity or selection. QTLs could be pyramided with other low-effect QTLs through genomic selection to obtain cucumber cultivars with enhanced field resistance to viruses.

## Introduction

Cucumber, *Cucumis sativus L.,* belongs to the Cucurbitaceae family and was domesticated in northern India 3000 years ago [1]. Modern cucumber varieties derived from a succession of severe bottlenecks caused by domestication and intensive modern breeding, resulting in a loss of genetic diversity in the European genetic group [2]. Cucumber is an important crop that is characterized by its biological plasticity [3], which is why it is able to thrive when cultivated in a wide range of environmental conditions, from northern European heated glasshouses to Indian open fields [4]. Consequently, cucumber crops are hampered by multiple biotic stresses including aphids, a major crop pest due to the damage it causes via herbivory and as a vector for numerous virus species [5, 6]. Two principal mechanisms are involved in aphid-borne virus transmission. Some viruses, such as the cucumber mosaic virus (CMV), are non-persistent in aphids; i.e., virus particles may be acquired by the aphid when it punctures an infected plant, become stuck in the insect’s mouthpieces and subsequently released and lost when the aphid bites/tastes another plant [7]. Conversely, some other viruses such as the cucurbit aphid-borne yellows virus (CABYV) are persistent in aphids*.* In this case, virus particles are ingested by the insect when it feeds on an infected plant, are stored in its salivary glands and subsequently transmitted to other plants through the fascicular phloem when the aphid feeds [8]—the aphid thus remains viruliferous [9]. So far, persistent virus damage has been successfully managed through pesticide control of the vector populations, which is why research on crop resistance to persistent viruses has been secondary as compared to non-persistent viruses [10]. However, recent consumer interest in more environment-friendly agriculture has given rise to pesticide reduction policies, thereby resulting in the resurgence of damage caused by persistent transmitted viruses such as CABYV in cucumber crops in France [11], and more recently in northern Europe [12, 13].

Quantitative trait loci (QTL) controlling plant virus resistance in vegetable crops have mainly been mapped by screening a diversity panel of the species to identify a potential resistance donor for subsequent segregant analyses [14]. Resistance genes identified in this way have therefore been restricted to the genetic diversity of the two parents of the segregating population. Genome-wide association studies (GWAS) directly use data collected on a diversity panel and have resulted in successful mapping of several plant virus resistance genes in various species [15]. GWAS is a statistical approach geared towards associating a phenotypic variation with a genetic variation such as a single nucleotide polymorphism (SNP). GWAS is designed to map quantitative traits and is hence able to map several low-effect QTLs that are usually involved in quantitative resistance, provided that resistant alleles are relatively frequent in the target population [16]. Post-GWAS analyses such as the local score approach (LSA) or LD decay analysis could complement GWAS so as to be able to map low-effect QTLs and generate insight into the resistance architecture [17].

Trials on phenotype resistance to viruses have often been performed in controlled conditions via mechanical seedling inoculation and symptom severity scoring [15]. This protocol has major spatiotemporal and cost-effectiveness advantages but does not reflect the complexity of plant virus interactions in the crop system. Indeed, abiotic stresses (temperature) or a combination of biotic stresses (co-inoculation with two different virus species, vector pressure, etc.) may lead to resistance being overcome in crop field conditions [18, 19]. Moreover, controlled persistent virus inoculations are tricky to perform as the vectors must be involved in the inoculation process. There is substantial literature on testing plant virus resistance in open field conditions with row crops such as maize, rice, and wheat [15]. Symptom severity notation is difficult in open field conditions because several virus species can induce the same symptoms that even experienced virologists cannot differentiate. In this scenario, only molecular virus detection methods such as ELISA or PCR are efficient for assessing the presence of a specific virus species in an open field crop.

In this study, we focused on two viruses featuring different characteristics. First, CMV, which is a highly polyphagous virus for which cucumber resistance has been described as being controlled by multiple quantitative loci when the virus was mechanically inoculated and monitored in insect proof glasshouses [20, 21]. Resistance to CMV was shown to be either monogenic or polygenic. GWAS after CMV mechanical inoculation revealed three QTLs localizing on chromosome 2, 5, and 6. The QTL on chromosome 5 colocalized with the RNA-dependent RNA polymerase 1b (RDR1b) gene, which confers cucumber resistance to CMV when silenced [22, 23]. Moreover, the eukaryotic initiation factor 4E (EIF4E) gene on chromosome 1 modified by CRISPR-cas9 conferred resistance to CMV [24]. Second, CABYV that is a virus that infects few plant species and for which resistance has to our knowledge never been studied in cucumbers. We sought to detect QTLs reducing CABYV and CMV epidemics in a large panel of cucumbers grown in open field conditions. We also mapped cucumber attractiveness for aphids so as to gain insight into the vector’s impact on the infection lag [25, 26]. Multiloci GWAS and an LSA, as recently proposed to detect low-effect QTLs [27], were used to decipher these QTLS.

## Results

### CABYV and CMV heavily infected cucumbers in the crop field test

Two hundred and fifty-six lines were grown in open field conditions. The presence of CABYV and CMV was tested by ELISA in each plant sixfold throughout the crop cycle as long as the plants were alive and CABYV- or CMV-negative. A total of 1575 plants were tested, representing 4790 collected samples and 8853 ELISA tests (Supplementary Table 1).

**Figure 1:**
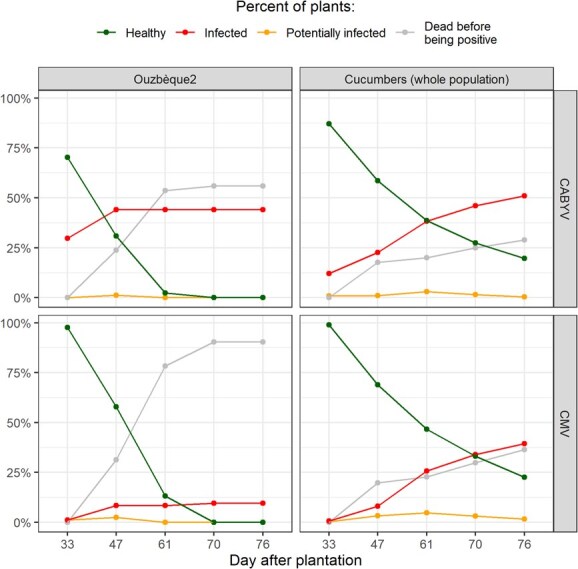
CABYV and CMV infection patterns in susceptible Ouzbeque2 melons and in the 256 lines at the population scale. The grey line represents the number of plants that were dead before being tested positive to the studied virus

CABYV and CMV were detected as of the first sampling date (33 DAP), and newly infected plants were identified up until the last sampling dates (76 DAP). We did not observe any specific initial infection sites in the crop field (Supplementary Fig. 1), thereby indicating that there was spatially homogeneous infection. ‘Ouzbeque2’ melon plants, which were regularly distributed in the field for control (Supplementary Fig. 2), were more frequently infected by CABYV than CMV. All ‘Ouzbeque2’ plants were infected by CABYV at 61 DAP and displayed intense yellowing or were even dead (Fig. 1, Supplementary Fig. 3). CABYV also infected the cucumber population sooner and more intensively than CMV, resulting in 81.9% CABYV- versus 65.8% CMV-infected cucumbers at 75 DAP (Supplementary Table 1).

One hundred and twenty-eight genotypes had enough live plants remaining at the end of the trial for epidemiological parameter inference. This population subset combined cucumbers of different types. Coinfection was observed in 34% of the plants, representing 109/128 co-infected genotypes (Supplementary Table 2). Nineteen genotypes were infected by CMV or CABYV, while only five of them were CABYV-free and 14 were CMV-free. No accessions were free of both viruses. Moreover, we used a data subset from Monnot et al. [21] to compare the resistance levels to CMV observed in crop fields with those observed after controlled inoculation and growth in glasshouses in the same lines (Supplementary Table 2). Briefly, in the study of Monnot et al., the population was grown in a greenhouse, mechanically inoculated 21 days after sowing and symptom severity was visually scored [1—no symptoms—to 9—highly susceptible]. Each accession was declared susceptible, intermediate resistant or resistant according to the criteria presented in Table 1. One accession that was considered as being highly resistant in open field conditions was considered susceptible in controlled conditions. Conversely, 25 lines considered as being highly resistant in controlled conditions had a high maximum CMV infection rate in crop field conditions.

### Aphid attractiveness variable among 149 cucumber lines

Aphid attractiveness was estimated on the basis of a leaf disk test of 152 lines (Supplementary Table 2) whereby aphids were allowed to choose among leaf disks of four different genotypes. Aphid attractiveness was simulated with each repetition data and with the combined raw data of both repetitions. Simulations with the combined data; i.e. each genotype was confronted to five other genotypes, had a Pearson correlation of 0.6 relative to the simulations obtained from each single repetition data. The most attractive cucumber genotypes belonged to the pickle horticultural group, while the least attractive were mostly beit-alpha cucumbers (Supplementary Table 2).

### Selection of traits for GWAS and their distribution within cucumber genetic diversity

We identified seven direct traits from ELISA CABYV and CMV field sample data, including the first infection date and virus incidence at six dates; and four derived traits, i.e. AUDPC and three Gompertz parameters. The AUDPC and Gompertz curves reflect the disease dynamics. In growth chambers, we analyzed the attractiveness to *A. gossypii*, which we expected to be a factor in the virus incidence in crop fields. Moreover, we used the symptom severity for CMV genotypes reported in Monnot et al. [21].

**Figure 2:**
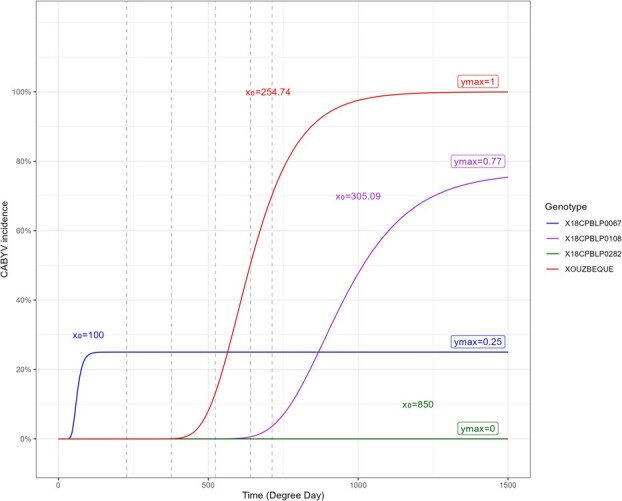
Four examples of Gompertz curves based on the CABYV incidence for four genotypes simulated on 1500 degree-days (~5 months). The dashed grey vertical lines represent the sampling dates. The Gompertz curves were described with three parameters: x_0_ (the infection lag), ymax (maximum infection rate), and K (maximum epidemic growth). Four reaction patterns were observed: full resistance (X18CPBLP0282), early infection but low maximum rate (X18CPBLP0067), late infection and high maximum rate (X18CPBLP0108) and full susceptibility, i.e. early infection and high maximum rate (XOUZBEQUE)

Therefore, we obtained a total of 22 traits and the complete correlation matrix of these traits highlighted two correlation spots: CABYV and CMV incidence traits (Supplementary Fig. 4). The pair of traits CABYV infection lag and attractiveness to *A. gossypi* were not correlated, whereas they could have been associated from a biological standpoint since *A. gossypii is* a major CABYV vector (Supplementary Fig. 4). Surprisingly, the Gompertz parameter K was not correlated with the other Gompertz parameters for CABYV or CMV. The unbalanced number of plants per accession actually did not enable us to reliably infer this parameter. Therefore, we selected six traits out of the 22 for GWAS. Two were obtained from tests conducted in controlled conditions; i.e. cucumber attractiveness to aphids and CMV symptom severity scores after mechanical inoculation. The four other traits were derived from the field test, i.e. the maximum infection rate and the infection lag (Gompertz parameters) for CABYV and CMV. We used two Gompertz parameters rather than AUDPC to quantify the disease patterns because AUDPC was unable to accurately capture the different epidemiology kinetics that we observed (Fig. 2). One pair of selected traits still had high correlation coefficients, i.e. CMV infection lag and CMV maximum infection rate (−0.56) (Supplementary Fig. 4). The CABYV and CMV maximum infection rates revealed an excess of highly susceptible accessions, while the other traits had more conventional Gaussian distributions (Supplementary Fig. 5).

The five-trait set related to CABYV and CMV resistance was obtained for 24 Long Dutch, 22 Beitalpha, 39 Pickles, 27 Slicers, and 16 landraces from the Crop Science internal collection, and traits related to attractiveness were obtained for 24 more genotypes (9 Long Dutch, 6 Beitalpha, 4 Pickles, 1 Slicer, and 4 landraces). Genotype phenotypic variability was plotted for each trait within the nine genetic groups (V1 to V9) defined in Monnot et al. [21] for the 256 genotype population (Supplementary Fig. 6). The V1 group contained Long Dutch, the V2 group contained mainly Asian landraces, the V3 contained European type landraces, the V4 and V5 contained Pickles, the V6 and V7 contained Slicers and the V8 and V9 Beitalphas. Some traits, such as aphid attractiveness and the CMV maximum infection rate, displayed homogenous phenotypic variance within each genetic group. The CMV symptom severity scores were contrasted: all accessions from the V3 genetic group were susceptible, while V2, V4, V5, V8, and V9 genotypes were highly resistant (Supplementary Fig. 6). The CABYV maximum infection rate was lower for V5 genotypes, and for admixed accessions to a lesser extent than for other groups.

**Table 1:** Distribution of genotypes in three classes of resistance levels to CMV according to their score in open field (column) and glasshouse (row) conditions.

		Open field resistance levels	
		Susceptible	Intermediate	Resistant
Glasshouse resistance levels	Susceptible	13	2	1
	Intermediate	33	7	8
	Resistant	25	14	25

An accession was considered resistant in the GH when its symptom severity score was <3, and susceptible when the score was >7. An accession was considered resistant in open field conditions when the maximum infection rate was <35% and as susceptible when the maximum infection rate was >75%.

### GWAS successfully unravelled multiple QTLs for the different traits

The six traits were analyzed by an MLMM GWAS with different models, including kinship (K) and/or genetic group assignment to five or nine groups (Q5 or Q9). We selected the model including both kinship and the nine genetic groups (KQ9) since the resulting *P*-values generated a curve that best fits the bisector on the QQplot regardless of the traits (Supplementary Fig. 7). The *P*-values collected in the first step of MLMM were used for an LSA to map low-effect QTLs, and we used the genomic control value to limit false positives.

The most significant peaks were detected by both MLMM and LSA, while less significant QTLs were detected by only one of the two methods. We detected a total of 35 QTLs, with 10 detected by both methods, 2 exclusively by MLMM, and 23 exclusively by LSA (Supplementary Table 3). Four to nine QTLs were detected per trait.

### CABYV epidemics were controlled by at least four loci in cucumbers

**Figure 3:**
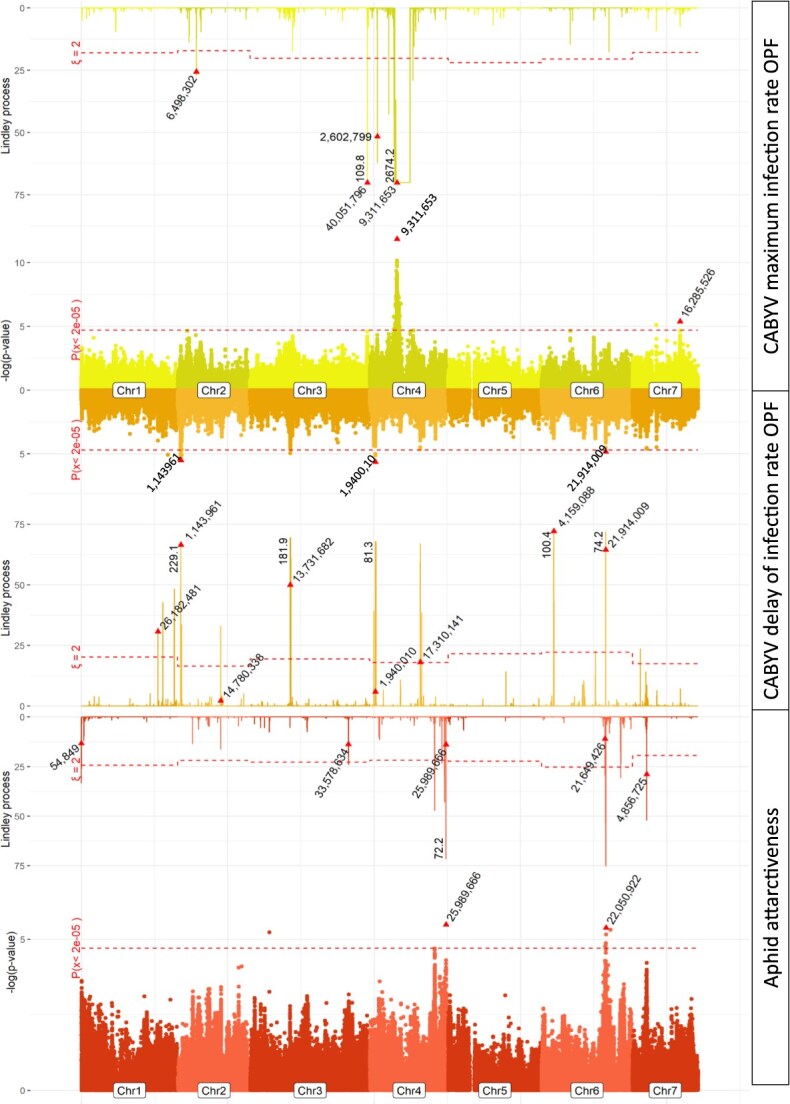
QTL mapping for the CABYV maximum infection rate and infection lag in open field conditions (OPF) and aphid attractiveness. Dots refer to MLMM analyses and bars are for local score analyses. The red triangle represents the top SNP of each QTL and its position. The dashed line represents significance thresholds: for the MLMM Bonferroni threshold according to the number of independent SNPs calculated by Gao et al. [28]. For the Lindley process, some bars were truncated for readiness but the actual value is displayed, the dashed line represents the genomic control value.

We detected five QTLs for the CABYV maximum infection rate (Fig. 3 upper part, Supplementary Table 3). A highly significant 2 Mb long QTL was detected on chromosome 4 by both methods. Its top SNP had an MAF = 0.24 and the resistant allele was heterogeneously distributed among the nine genetic groups—two features that made this QTL very interesting. This QTL reduced the maximum infection rate by 42% (Supplementary Table 3). Two QTLs that were less significant than the previous one, but which showed about the same average difference in maximum infection rate, were mapped on chromosome 7 by MLMM and on chromosome 4 by LSA. Two low-effect QTLs were mapped by LSA on chromosomes 2 and 4. Eight QTLs were detected for the CABYV infection lag (Fig. 3 middle part, Supplementary Table 3), including three detected by both MLMM and LSA at the beginning of chromosomes 2 and 4 and at the end of chromosome 6. The last QTLs for CABYV infection lag were mapped by LSA on chromosomes 1, 2, 4 and 6. The three QTLs mapped by MLMM displayed a difference ranging from 50 to 200 degree-days with regard to the CABYV infection lag (Supplementary Table 3), corresponding to 6 to 22 days in spring in southeastern France. Overall, four QTLs were mapped by both methods, one for maximum infection rate and three for infection lag. The highly significant 2 Mb long QTL detected on chromosome 4 for the CABYV maximum infection rate might also influence the infection lag.

We screened for putative colocation of those QTLs with attractiveness to *Aphis gossypii*, a major CABYV vector. Five QTLs were mapped for cucumber attractiveness to aphids (Fig. 3 lower part, Supplementary Table 3). One QTL was mapped on chromosome 6 by both methods and importantly colocalized with the major effect QTL for CABYV infection lag. However, the MAF of the top SNP was low (6.9%) and the repulsive allele was only present in genetic group 1 containing Long Dutch lines (Supplementary Table 3). Another QTL mapped on chromosome 4 was less significant but provided more contrasted phenotypes—but higher standard deviation—and a better balance in allele frequency among genetic groups.

### Loci controlled CMV epidemics independently of those controlling symptom expression

**Figure 4:**
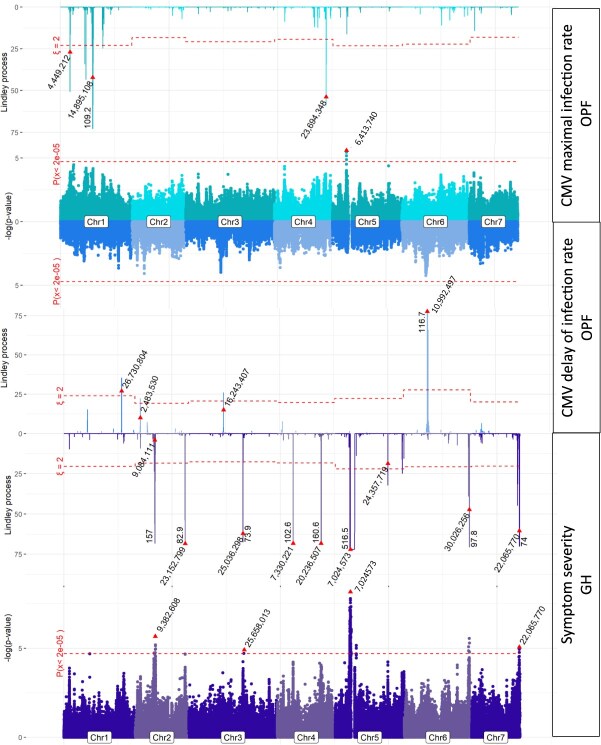
QTL mapping for CMV maximum infection and infection lag in open field conditions (OPF) and symptom severity in glasshouses (GH). The red triangle represents the top SNP of each QTL and its position. In the Miami plots (in the middle for the CMV epidemic traits and in the lower part for CMV symptoms), the dashed line represents the Bonferroni threshold according to the number of independent SNPs calculated by Gao et al. [28] per trait. For the Lindley process (top and bottom for CMV epidemic traits and top for CMV symptoms), the dashed line represents the genomic control value. The *y-*axis on the Lindley process plot was truncated for readiness and the maximum value on the *y-*axis is indicated on the Lindley peak

Four QTLs were detected for the maximum CMV infection rate in field conditions (Fig. 4 upper part, Supplementary Table 3) by MLMM or LSA. A QTL was mapped by MLMM just upstream of an SNP gap on chromosome 5, while the allele frequency was unbalanced between genetic groups, with both homozygous states only observed in groups 2 and 5 (Supplementary Table 3). QTLs had an effect in reducing the infection rate by around 30% except for the second QTL mapped on chromosome 1 by LSA for which the infection rate was only reduced by 7%, with a high standard deviation (Supplementary Table 3). Regarding the CMV infection lag, the most significant QTL was a large 4 Mb QTL (Fig. 4 middle part, Supplementary Table 3) mapped on chromosome 6 by LSA, while the average difference between the two homozygous groups was 70 degree-days, corresponding to 8 days in spring in southeastern France. Three other QTLs were mapped on chromosomes 1, 2, and 3.

We screened for putative colocation of those QTLs with QTLs controlling symptom severity in controlled conditions after CMV mechanical inoculation. Overall, we mapped nine QTLs, four of which were mapped with both MLMM and LSA (Fig. 4 lower part, Supplementary Table 3). Remarkably, a QTL on chromosome 5 had a top SNP located 600 kb downstream of the top SNP for CMV maximum infection rate in field conditions. Despite being physically close, the two resistant alleles from the two top SNPs did not display the same pattern with regard to frequency among genetic groups (Supplementary Table 3), suggesting that they belonged to different LD groups.

Therefore, among the eight QTLs controlling epidemics of CMV, only one colocalized with the nine QTLs reducing symptoms after mechanical inoculation. This suggested that both traits were genetically independent. Two hypotheses might explain this independence; i.e. other QTLs reducing symptoms after mechanical inoculation were either related to tolerance (no symptoms in CMV-infected plants) or were inefficient against the virus isolates occurring in the field.

### Local LD and haplotype studies help to: i/ gain insight into genetic blocks and thus the recombination history, and ii/ dissect resistance clusters

**Figure 5:**
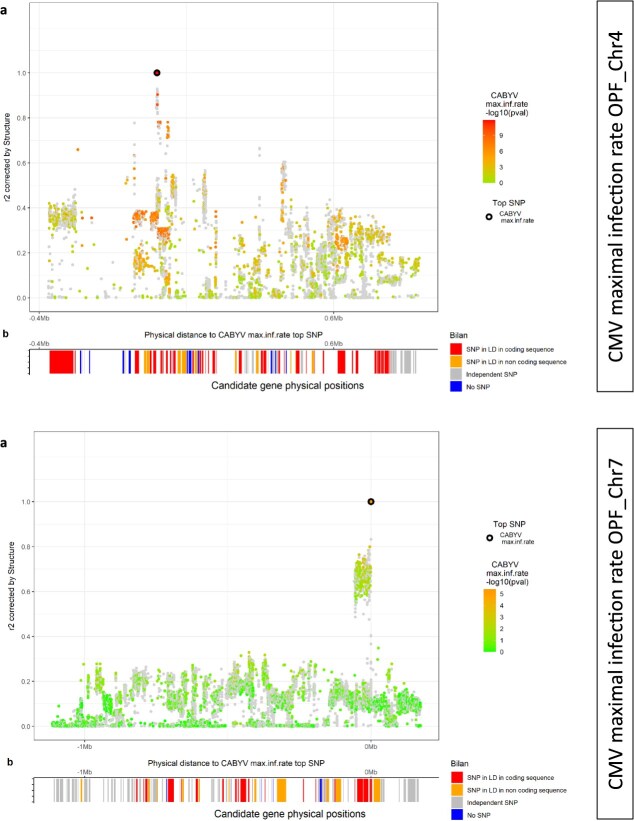
Local LD study on the QTL controlling the CABYV maximum infection rate on chromosome 4 (upper part) [8.6–10.6 Mb] and on chromosome 7 (lower part). A. each dot represents an SNP, on the *y*-axis the *r*^2^ with the top SNP of the QTL represented by a dark dot. Grey dots represent SNPs with at least one missing data that were not studied by GWAS. The reddest dots reflect greater significance. B. Classification of genes located in the QTL interval according to the presence of SNPs in LD with the top SNP in coding or non-coding sequence

When considering all QTLs, two LD decay patterns were observed, as illustrated in Fig. 5. The first pattern concerned large QTLs with slow and regular LD decay (LD of each SNP with the top SNP inferred by the *r*^2^ corrected by the structure). This was the case for the QTL_CABYVmaxinfrate_ch4_ (Fig. 5, upper part). The second LD decay pattern also concerned large QTLs, but in this case there was sharp LD decay around the top SNP. This pattern was observed for QTL_CABYVmaxinfrate_ch7_ which presented a clear LD block of 150 Mb (Fig. 5 lower part), or for the cluster on chromosome 6 pooling QTLs for CABYV infection lag and aphid attractiveness (Supplementary Fig. 8).

We assessed virus resistance in a panel that mainly contained elite lines and therefore expected that recently introduced virus resistances would be located in genomic areas where there was sharp LD decay, unlike the pattern regarding resistance that had been introduced early in breeding programs. This suggested that LSA would be particularly interesting for detecting low-effect QTLs that had been recently introduced in breeding programs via serendipity or selection.

Genes present in the QTLs were identified and SNPs in coding and non-coding sequences (predicted in the Cornell Chinese Long (CCL) V3 annotated genome [29]) were spotted (see Figs 4 and 5b and Supplementary Fig. 8B for examples) [30]. All QTLs contained at least one gene with an SNP in a coding sequence being in LD with the top SNP. The number of candidate genes ranged from 4 to 208 (Supplementary Table 3). Focusing on the nine QTLs detected by both methods, we identified the genes which both had an annotated function and were included in a genomic area in LD with the top SNP (Supplementary Table 4). For attractiveness, we identified 140 genes with at least one SNP in an exon and in LD with the top SNP, with one of them related to disease resistance (CsaV3 4G03640). For the CABYV infection lag, we identified 88 genes including CsaV3_6G033980 related to disease resistance while for CABYV maximum infection rate we identified 50 genes, none of them with a remarkable function. For CMV symptom severity we retrieved 191 genes including CsaV3_5G010740, a putative Eukaryotic translation initiation factor 1A and CsaV3_3G028850, a Vesicle-associated membrane protein. Almost all QTLs displayed GO enrichment, while two QTLs were particularly enriched in GO related to disease resistance. The QTL for aphid attractivity on chromosome 7 was enriched in genes involved in extracellular space functions (14%), and the QTL for the CABYV maximum infection rate on chromosome 4 was enriched in genes involved in the cellular response to stress (15%) (Supplementary Table 5).

**Figure 6:**
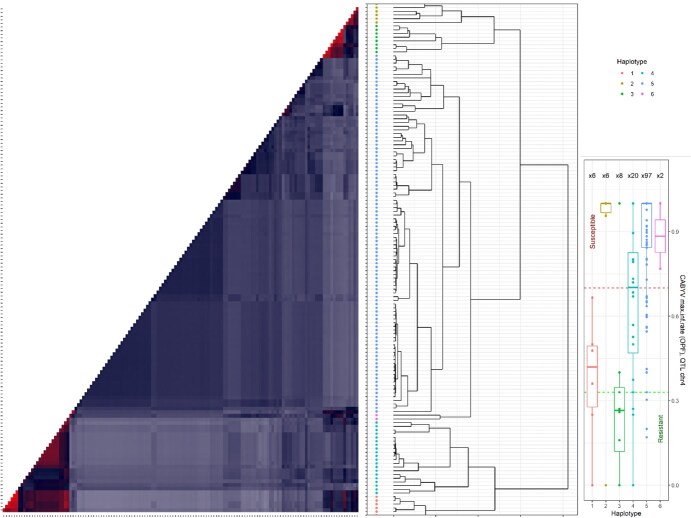
Haplotype study in the QTL for CABYV maximum infection rate on chromosome 4. The clustering of local kinship represented by the heatmap on the left and the dendrogram highlighted the presence of several haplotypes represented in the heatmap on the right. Boxplots represent the phenotypic variability within each haplotype

Haplotypes present in a QTL genomic area; i.e. when the LD with the top SNP was below 0.2, were designed using local kinships for all traits deriving from field experiments and aphid trait (haplotypes for CMV traits obtained in greenhouse were presented in Monnot et al. [21]. The QTL_CABYVmaxinfrate_ch4_ contained at least six haplotypes in the panel (Fig. 6). Two of them, the haplotypes one and three, displayed the lowest infection rates. The haplotype 1 pooled individuals from the second group of genetic structure including Asian landraces and pickles which have a general good level of resistance to several virus species (Supplementary Fig. 6). The other lines with the haplotypes 1 and 3 are six admixed accessions according to structure groups, and few Pickles, Slicers, and Beitalphas according to horticultural groups. Other QTLs revealed multiple haplotypes without clear segregation of extreme phenotypes.

## Discussion

**Figure 7:**
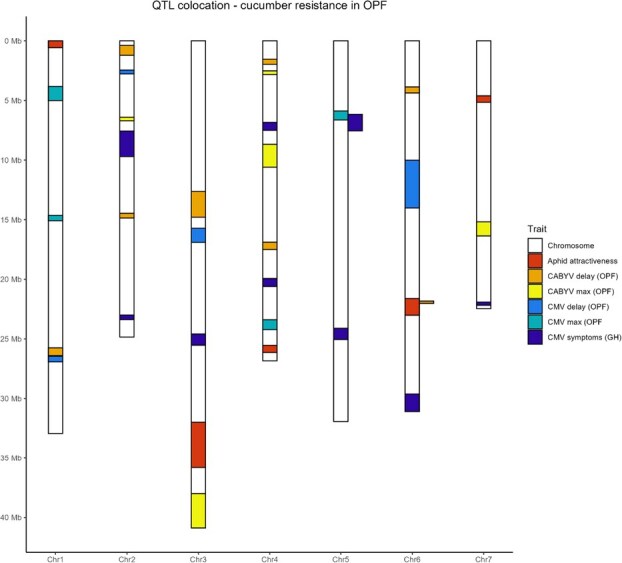
Location of 35 QTLs on the cucumber genome (OPF = open field trial, GH = greenhouse trial)

In this study, which aimed at identifying QTLs that could reduce CABYV and CMV epidemics in cucumber fields, we mapped 35 QTLs related to a virus phenotype or a virus-vector phenotype (Fig. 7).

We set up a field experiment design to manage the effective inoculation of cucumber plants by both viruses, distributing susceptible melon regularly in the field, while confirming the presence of both viruses through ELISA tests. As expected, all surviving check plants were positive to both viruses at the end of the experiment. This strongly suggested that all plants had been visited by viruliferous aphid vectors. We used two methods to detect QTLs, i.e. MLMM and LSA, with the latter being carried out specifically to identify low-effect QTLs [17]. Accordingly, LSA enable the mapping of low-effect QTLs and confirmed QTLs that have barely reached the significance threshold using conventional MLMM GWAS. Notably, we showed that both virus epidemics were controlled by 8–13 QTLS, which highly suggested that strong resistance genes were not the primary contributors to resistance against CABYV or CMV. We mapped these QTLs using a cucumber panel of 131 elite lines and 18 landraces. QTLs that had been recently introduced in the elite panel were expected to be large and flanked by sharp LD decay. For several QTLs for CABYV resistance, the decrease in local LD decay was shorter than that of CMV QTLs, thereby reflecting the more recent introduction of some QTLs controlling CABYV in the breeding panel.

Surprisingly, few published studies have focused on plant interactions with CABYV despite its prevalence and the significant cucumber yield decreases noted in CABYV infection cases [11]. Unlike potyviruses or other viruses such as CMV, the major symptoms of CABYV are flower abortion, which can be mistaken for poor yield observation, and old leaf yellowing, that can be easily confused with aging symptoms, nitrate deficiency or fungal attacks. The scarcity of previous studies on CABYV resistance could be attributed to the difficulty in using symptomatology, that is typically used for genetic resistance studies [15]. We identified thirteen QTLs controlling CABYV epidemics, with eleven being supported by several significant SNPs that formed a peak on the Manhattan plots. These QTLs delayed the epidemics by 6 to 22 days in the field test and reduced the maximum infection rate by around 40%. At the end of the trials, five accessions were completely free of CABYV, demonstrating that there were diverse and quite frequent sources of resistance in the tested panel. For example, QTL_CABYVmaxinfrate_ch4_ is a wide favorable allele that is prevalent in different genetic groups (50% in Asian landraces, 30% in a Beitalpha group, 18% in a pickle group, but only 3% in Long Dutch group). Interestingly, QTL_CABYVmaxinfrate_ch4_ colocalized with *dm4.1* and *pm4.1,* two important QTLs that, respectively, control cucumber resistance to downy and powdery mildew [31]. CABYV resistance could be introduced serendipitously by breeders when seeking to introduce resistance to powdery and downy mildew, especially the latter which produces CABYV-like symptoms. CABYV resistance could also be serendipitously introduced when breeding for yield, but we were unable to check this hypothesis since we did not find any previous reports on QTLs controlling yield or flower abortion.

Cucumber resistance to CMV has been previously described as being controlled by multiple quantitative loci [20, 21] when mechanically inoculated and monitored in insect proof greenhouses. In this study, we identified eight QTLs that controlled CMV epidemics in field conditions in southeastern France. Fourteen accessions out of the 149 in the panel were observed to be CMV symptomless in controlled conditions but had an infection rate > 70% in open field conditions. Notably, only QTL_CMV_ch5_ was shown to reduce both symptoms after mechanical inoculation and field epidemics. Several not mutually exclusive hypotheses could explain this discrepancy. First, accessions with high infection rates in the field but which were symptomless in controlled conditions could be tolerant to CMV. Accordingly, some of these 14 accessions did not display any mosaic symptoms in the field despite the high infection rates. Tolerance is an intriguing trait as it allows the plants to remain vigorous in the absence of selective pressure on the virus strains, thereby avoiding resistance breakage. However, tolerant and infected plants can act as a virus reservoir, thus potentially jeopardizing neighboring crops. A second hypothesis is that co-inoculation with another virus, such as potyviruses or CABYV, helped CMV overcome plant resistance. This phenomenon, known as CMV resistance breakage, has been described in cucumber crops co-infected with CMV and ZYMV [19]. Unfortunately, ZYMV prevalence was not assessed in this trial due to its rarity in the area. A third hypothesis is that there are differences in virus strains when comparing the greenhouse and field tests. Indeed, viruses display intraspecific genetic diversity in field conditions, and the different strains can target different plant resistance mechanisms [32]. More than 72 CMV isolates were detected following a sampling campaign in the French Mediterranean Basin in 2016 and 2017 [33], i.e. in the vicinity of our open field trial. In contrast, we mechanically co-inoculated two CMV strains in the glasshouse tests. Interestingly, QTLs reducing CMV epidemics—a trait that is currently not exploited in breeding programs—showed a shorter local LD decay pattern compared to QTLs reducing symptoms—a trait that is commonly used in breeding programs. This suggested that they had been serendipitously introduced in the elite panel, as we discussed previously with regard to CABYV. QTLs reducing CMV epidemics contained some remarkable genes. QT L_CMV_GH_ch1_ was 4 Mb away from eukaryotic translation initiation factor 4e, a gene involved in resistance to CMV when modified [24]. Interestingly, QTL_CMV_GH_ch1_ contained CsaV3_5G010740, a putative eukaryotic translation initiation factor 4 g (Supplementary Table 4). Moreover, QTL_CMV_GH_ch1_ contained a calmodulin gene, which was shown to be involved in plant immunity [34], and a vesicle associated protein gene, CsaV3_3G028850, that could act like VPS4 for CMV resistance in melon [35]. Furthermore, a QTL mapped on chromosome 6 was only 1 Mb away from *cmv.6.1*, a QTL of resistance to CMV that previously mapped in cucumber in a segregating population [36].

Our study was focused on two types of viruses differing in their transmission mode: persistent CABYV and non-persistent CMV. The former is transmitted by a limited number of aphid species, notably *A. gossypii*, the primary aphid species colonizing cucurbit fields. Conversely, CMV is transmitted by hundreds of aphid species. This suggested that the plant/*A. gossypii* interaction played a role in CABYV epidemics but not necessarily in those of CMV, unless *A. gossypii* was the primary CMV vector in the field trial. In our laboratory experiments, we assessed aphid attractiveness using the *A. gossypii* clone most prevalent in southeastern France [37]. Aphid attractiveness was evaluated in a choice test that mirrored field conditions where all accessions were available to aphids. Interestingly, attractiveness to *A. gossypii*, a vector for both CABYV and CMV, was more closely correlated with the CABYV infection lag than CMV (Supplementary Fig. 5). We identified five QTLs reducing aphid attractiveness, two of which were detected by both MLMM and LSA. Remarkably, one QTL for aphid attractiveness colocalized with CABYV infection lag on chromosome 6. This suggested that the QTL for CABYV infection lag on chromosome 6 could be inefficient when a unique cultivar is grown and therefore, where no choice is offer to aphids. A second QTL for aphid attractiveness on chromosome 7 was remarkable, given that it was enriched in genes involved in extracellular space functions (Supplementary Table 5), a space in which stylets penetrate as once aphids puncture a plant [38]. Finally, QTLs reducing attractiveness did not colocalize with the only previously detected by bulk analysis during cucumber infestation by *A. gossypii* Glover [5]. This QTL was mapped in the distal area of chromosome 5 (26.6 Mb).

In this study, we mapped different QTLs involved at different phases of virus epidemics in the field. Finally, pyramiding QTLs reducing epidemics could definitely help to enhance resistance levels and sustainability [39]. Pyramiding all of these low-effect QTLs would be a challenge for breeders, so genomic selection could offer an effective alternative [40].

## Materials and methods

The cucumber population used to test CABYV and CMV infections was the same as that tested in glasshouse conditions (GH) in Monnot et al. [21]. It pooled 256 lines and was grown in an open field trial (OPF) in the vicinity of Saint-Andiol (France), a cucumber growing area where both viruses have been the most prevalent virus species [41]. The trial consisted of two randomized complete blocks of five plants, representing ten plants per genotype. Viruses were naturally inoculated by wild aphid populations. The ‘Ouzbeque2’ melon variety, which develops intense leaf yellowing when infected by CABYV, was used as a susceptible control [42] (Supplementary Fig. 3). Twenty-two five-plant plots of ‘Ouzbeque2’ were regularly included in the experimental design so as to spatially promote homogenous infection spreading (Supplementary Fig. 2). Sampling was initiated when the leaves of the ‘Ouzbeque2’ melon control began yellowing.

### An efficient ELISA protocol to infer the virus incidence in a large cucumber population

CABYV and CMV infections were assessed in series of six double-antibody ELISA tests at 33, 40, 47, 61, 70, and 76 days after plantation (DAP). We expected to detect CABYV on older leaves, while expecting CMV to prevail on younger leaves [11]. Therefore, plants were sampled by collecting 20 cm^2^ of the second and the fourth leaves from the apex, which jointly gave around 2 g of leaf material. A single branch per plant was labelled and sampled throughout the trial. Samples were ground in 4 ml of extraction buffer and tested for both CABYV and CMV presence via the SEDIAG testing kits [43]. Antibodies were diluted at 0.5/100 to decelerate the revelation process due to the high number of tests to perform per day. ELISA plates were deposited the same day as the sample collection. For the same reason, the coating step was performed three days before the sampling date and plates were frozen at −20°C. Samples were declared positive when their absorbance was threefold above that of the negative control sample (healthy ‘Aramon’ cucumber plants), potentially infected when their absorbance was twofold above that of the negative control sample, and otherwise negative according to Sutula *et al.* [44]. All plants were sampled at the first phenotyping date, and then plants were no longer sampled once they were declared CABYV- and CMV-positive.

### Virus incidence to derive the epidemiological parameters

First, CABYV and CMV incidences were, respectively, calculated per genotype at each date (on a scale of 0%–100% infected plants). Many plants died before the end of the trial without being tested positive to one of the tested viruses due to the hot windy conditions that prevailed in the vicinity of Saint-Andiol, southern France, in the spring of 2020. Consequently, only cucumber accessions with at least three phenotyped individuals at the end of the trial were kept for inference of the epidemiological parameters. This population subset pooled 128 genotypes. Several traits were derived from the qualitative ELISA tests per genotype: the first infection date, the area under the disease progress curve (AUDPC) based on the degree-day after plantation and a Gompertz curve were plotted for each accession that had at least one infected plant, using the following formula (Fig. 2):

$x(t)=K\times \mathit{\exp}\Big(\ln \left(\frac{x_0}{K}\right){e}^{-{y}_{max}t}$ ).with ${x}_0$ being the infection lag, $K$ being the maximum absolute growth rate, and ${y}_{max}$ being the maximum infection rate.

Parameters were set at ${x}_0=850$, $K=0$, and ${y}_{max}=0$ for accessions that had no infected plants.

### Plant attractiveness—a putative key component in plant virus resistance

A population subset of 152 lines was tested for its attractiveness to the *A. gossypii* CUC1 clone, i.e. the most frequent clone in the area [37], in a multiple-choice bioassay that mimicked the field conditions better than non-choice tests. Aphids were reared on the ‘Vedrantais’ melon variety a week before the test and were synchronized to get a cohort of uniform age [45]. Four plants per genotype were sown and nine leaf disks (2 cm dia.) were extracted 21 days later. Leaf disks from three randomly chosen genotypes were deposited with their upper leaf side on agar media (4 g/L of agar and 1 0 g/L of mannitol) in a closed box. Leaf disks from the CCL landrace, which is assumed to be aphid repulsive, were inserted between the leaf disks of genotypes to be tested (Supplementary Fig. 9). One apterous aphid was deposited on each leaf disk from the three tested genotypes (none aphid deposited on the CCL leaf disks). Boxes were stored for 24 h in a climate chamber at 24/18°C under a 16/8 h photoperiod. The number of aphids feeding on each genotype was assessed the following day. Moving or dead aphids were discarded from the analyses.

We hypothesized that the attractiveness of a genotype to aphids would be reflected by the number of detected aphids feeding on it. Conversely, a genotype with no, or less than the nine initially deposited, aphids feeding on it could be considered repulsive/less attractive than other genotypes present in the box. Attractiveness of a genotype is relative to the attractiveness of the neighboring phenotypes; therefore, we performed an experiment with two complete randomized block designs and attractiveness of each genotype being estimated by maximum likelihood using 5000 simulations.

The number of aphids on each genotype after 24 h was analyzed on the basis of two hypotheses: i/aphids acted independently and ii/aphid behaviors inside the box were statistically similar. Under these two hypotheses, the probability of observing ${n}_g$ aphids on genotype *g*, ${P}_g\left(x={n}_g\right)$*,* was described as follows:


$$ {P}_g\left(x={n}_g\right)={\left({p}_{gi}\right)}^{n_g} $$

where ${p}_{gi}$ denotes the probability of observing an aphid on the i.e. leaf disk of genotype *g* and was modelled as:


$$ {p}_{gi}=\frac{a_{ig}}{\sum{a}_{i,b}} $$

where ${\mathbf{a}}_{\mathbf{i}}$ attractiveness of the i.e. leaf disk, $\sum{\mathrm{a}}_{\mathrm{i},\mathrm{b}}$ sum of the attractiveness of all leaf disks from box b,${\mathbf{P}}_{\mathbf{i}}$ probability that an aphid would be on leaf disk *i* and $P\Rightarrow B\left(\begin{array}{c}k\\{}N\end{array}\right)$,$N\in [\![ 0.27]\!]$, the number of aphids per box, and $k\in [\![ 0.54]\!]$, the number of leaf disks per box.

By convention, $\sum_{g\in G}{a}_g=1$, with *G* being the number of genotypes tested, and with ${a}_g$ being estimated by maximum likelihood using 5000 simulations.

### A dense genotyped population

The genotyping and sequencing protocols are described in the supplementary data of Monnot et al. [21]. Briefly, ILLUMINA short reads were aligned on the CCL V3 reference genome, thereby generating a final matrix of 1.3 M SNPs for the 256 genotypes from Monnot et al. [21]. Quality filtering (heterozygosity <15%, missing data = 0%, minor allele frequency/MAF > 3.1%) reduced the number of SNPs available for GWAS to 570 738. We estimated the number of independent SNPs using the simple method of Gao [28] with a sliding 1 Mb window resulting in the identification of 17 970 (CMV maximum infection rate) to 24 679 (CMV mechanical inoculation) independent SNPs. The 570 k SNPs tested by GWAS thus adequately covered the genome. We used a subset of the genetic structure matrix calculated in Monnot et al [21]. because all groups were well represented in the panel subset. The VanRaden additive kinship matrix was recalculated on the population subsets.

### Detection of additive QTLs with multiloci GWAS: multiloci mixed linear models

QTLs controlling traits selected after correlation analyses were mapped by GWAS using five multiloci mixed linear models (MMLM), which considered the kinship effect and/or the genetic structure using five or nine ancestral groups: K, Q5, Q9, KQ5, and KQ9. In addition, we retrieved the phenotypic data for the population subset for resistance to CMV in GH available in Monnot et al. [21] and tested these traits with the same GWAS models. Briefly, the population was mechanically inoculated 21 days after sowing and symptom severity scores were visually attributed according to a 1 (no symptoms) to 9 (highly susceptible) notation scale.

MLMM is an iterative GWAS approach that selects the most significant SNPs from the previous steps and adds them as a fixed effect in the GWAS model of the following step. It enables the detection of additive SNPs.


$$ y={\mathrm{X}}_{\mathrm{M}}{\beta} +{\mathrm{X}}_{\mathrm{W}}{\nu} +g+e $$

where *y* is the phenotype vector; β is the vector of allelic effects; ${\mathrm{X}}_{\mathrm{M}}$ is the genotypic matrix of all accessions at the tested SNP; ν is the fixed effect vector, including the population genetic structure and top SNP, depending on the model and MLMM step; ${\mathrm{X}}_{\mathrm{W}}$ is the fixed effect incidence matrix; g is the random genotype effect vector; $\mathrm{g}\to \mathrm{N}\left(0,{\mathrm{K}{\sigma}}_{\mathrm{g}}^2\right)$; K is the kinship matrix (= I for Q5 and Q9 models); ${{\sigma}}_{\mathrm{g}}^2$ is the genetic variance; e is the error vector; ${\sigma}_e^2$ is the residual variance; and $\mathrm{e}\to \mathrm{N}\left(0,{\mathrm{I}{\sigma}}_{\mathrm{e}}^2\right)$ where g and e are assumed to be independent.

The significance threshold was set for each trait with a Bonferroni threshold corrected by the number of independent SNPs identified with the simpleM method. We selected the KQ9 models based on QQ-plots to identify top SNPs. QTLs were delineated by a local linkage disequilibrium approach and borders were set when no further SNPs with *r*^2^ > 0.2 with respect the top SNP was found.

### Detection of low-effect QTLs and candidate genes with a set of post-GWAS analyses

The LSA was initially developed to detect selection signatures by scanning forward and backward SNPs in the genome using *F_st_* parameters. It was successfully translated to detect low-effect QTLs by scanning SNPs in the genome using *P*-values collected by GWAS [17, 27]. We used ξ = 2 and calculated the genomic inflation factor ${{\lambda}}_{\mathrm{GC}}$. When ${{\lambda}}_{\mathrm{GC}}$>1.001, *P*-values were resampled to set the significance thresholds.

The local LD study, candidate gene research and haplotype analysis conditions are described in Monnot et al. [21] and were run for all QTLs. Briefly, local LD analysis relies on calculation of the LD corrected by the structure between the top SNP and all SNPs in a sliding window of 100 kb. Each gene containing at least one SNP in a coding or non-coding area with *r*^2^_S_ > 0.2 with respect the top SNP was considered as a candidate gene. For haplotype analyses, the local kinship matrices were clustered by hierarchical clustering. Haplotypes were defined based on kinship coefficients to pool resistant lines by potential resistance sources. Gene ontology enrichment was studied using Omicsbox software [46] by testing the most specific GO with an exact test and FDR = 5%.

## Supplementary Material

Web_Material_uhaf016

## Data Availability

Phenotyping data are in supplementary table file. Genetic data are in Monnot, S.; Cantet, M.; Mary-Huard, T.; Moreau, L.; Lowdon, R.; Van Haesendonck, M.; Ricard, A.; Boissot, N. Unravelling Cucumber Resistance to Several Viruses via Genome-Wide Association Studies Highlighted Resistance Hotspots and New QTLs. Horticulture Research 2022.
