# Supplementary material for: Genome-wide association studies to assess genetic factors controlling cucumber resistance to CABYV and CMV in crop fields and the attractiveness for their Aphis gossypii vector
Source: Hortic Res. 2025 Jan 14;12(5):uhaf016. doi: 10.1093/hr/uhaf016 (PMC11975396; doi:10.1093/hr/uhaf016)
Supplement: Web_Material_uhaf016 [file web_material_uhaf016.zip › CABYV_SupFigures_2024_R1.docx]

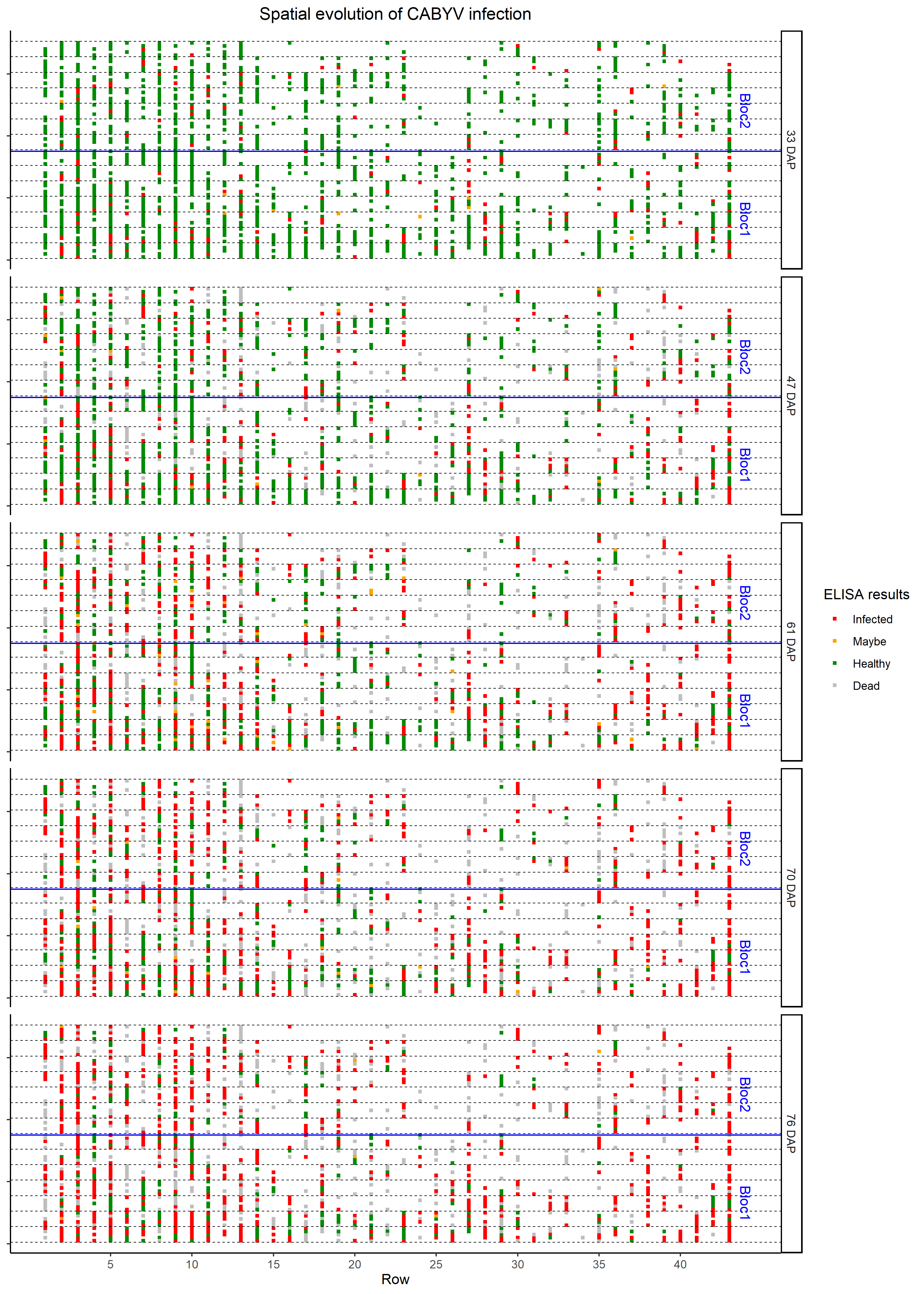


Supplementary figure 1: Spatial and temporal evolution of CABYV infection in the field.

Supplementary figure 2: Open field map, the two complete randomized bloc were oriented perpendicularly to the wind. Borders are composed by one ‘Ouzbeque 2’ melon variety plant which is the susceptible control. Orange plot represents 5 ‘Ouzbeque 2’ melon variety plants.


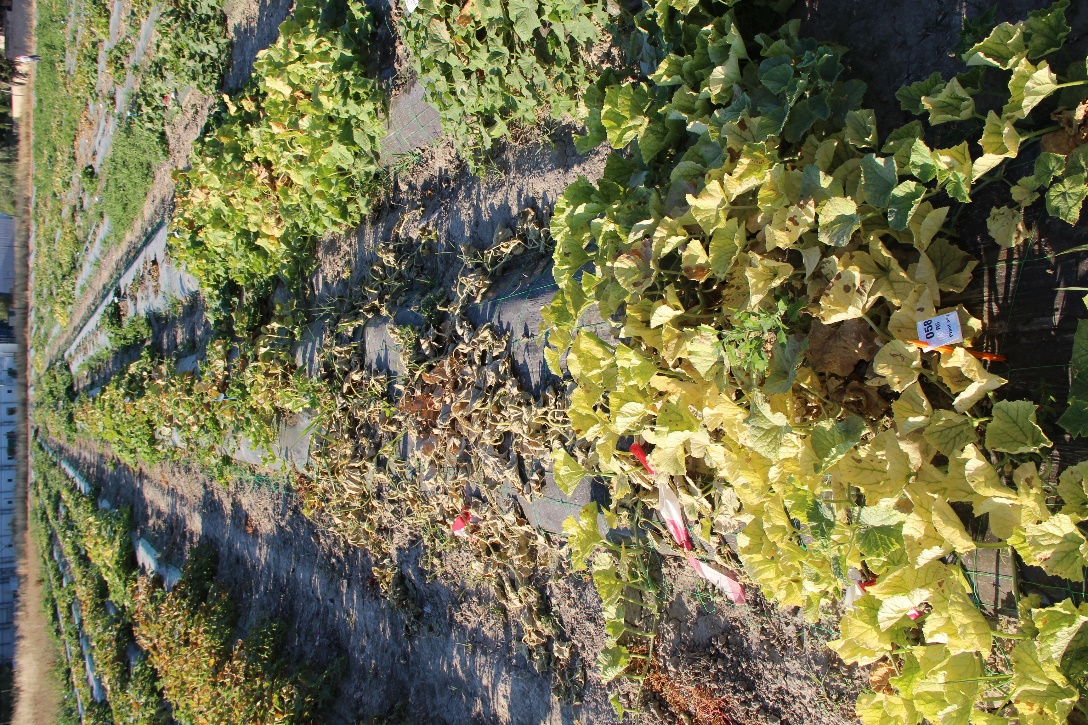


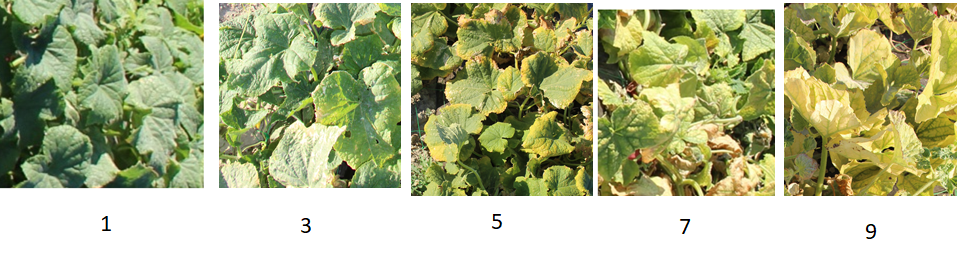


1 3 5 7 9

Supplementary figure 3: The melon variety ‘Ouzbeque 2’ displays intense leaf yellowing when infected by CABYV. Scale notation for yellowing, 1: dark green, 3: light green, 5: yellow spots on elder leave, 7: light yellow, 9: intense yellow


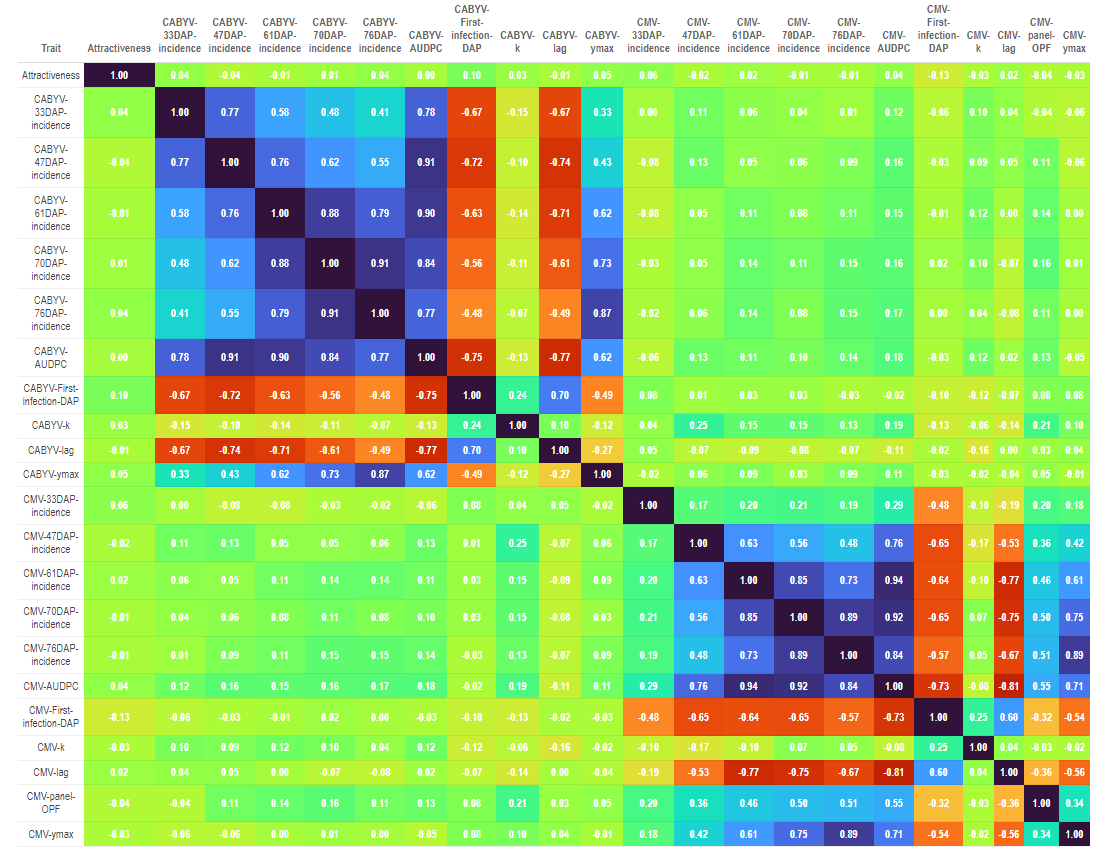


Supplementary figure 4: Correlation studies performed on the 22 phenotyping traits collected

Aphid attractivity simulated on one or two repetitions: Attractivity-combine/S1/S2

CABYV incidence single sampling: CABYV-33/47/61/70/76-DAP-incidence

CMV incidence single sampling: CMV-33/47/61/70/76-DAP-incidence

CMV or CABYV first infection: CMV/CABYV-first-infection-DAP

CMV or CABYV AUDPC: CMV/CABYV-AUDPC

CMV or CABYV delay of infection: CMV/CABYV-lag

CMV or CABYV maximal infection rate: CMV/CABYV-ymax

CMV or CABYV maximal infection speed: CMV/CABYV-k

CMV or WMV symptom severity (GH)


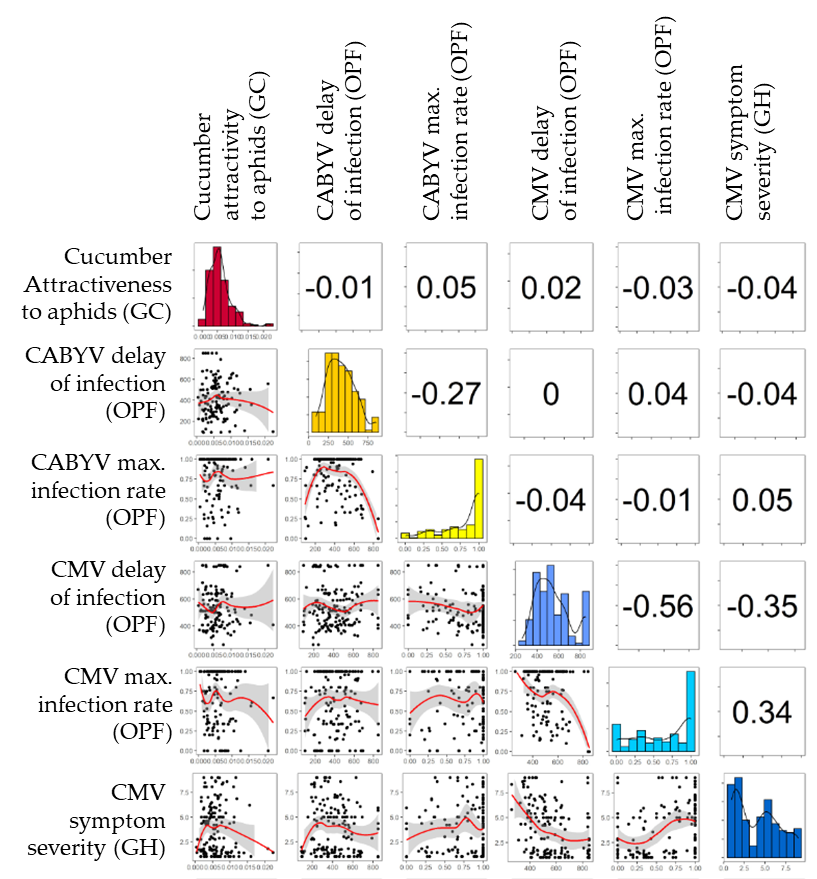


Supplementary figure 5: On the diagonal, phenotype distribution for the selected trait: Aphid attractivity calculated, CABYV delay of infection, CABYV maximal infection rate, CMV delay of infection, CMV maximal infection rate, CMV symptom severities scored in glasshouse, Upper part, Pearson’s correlation r^2^, lower, matching scatter plots with the regression curve in red.


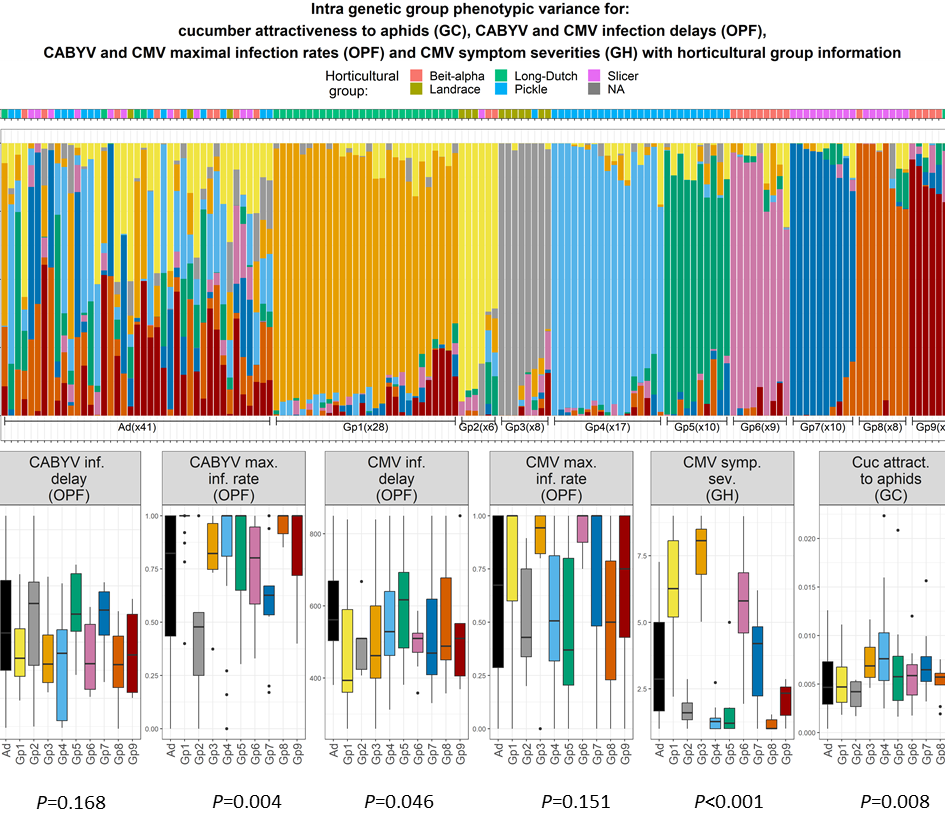


Supplementary figure 6: The nine genetic groups detected by sNMF in Monnot et al, 2022 are well represented in the panel subset, and each of them can be associated to a horticultural group. The phenotypic variance intergroup is stable or unbalanced according to the trait studied (*p* value from ANOVA analysis).


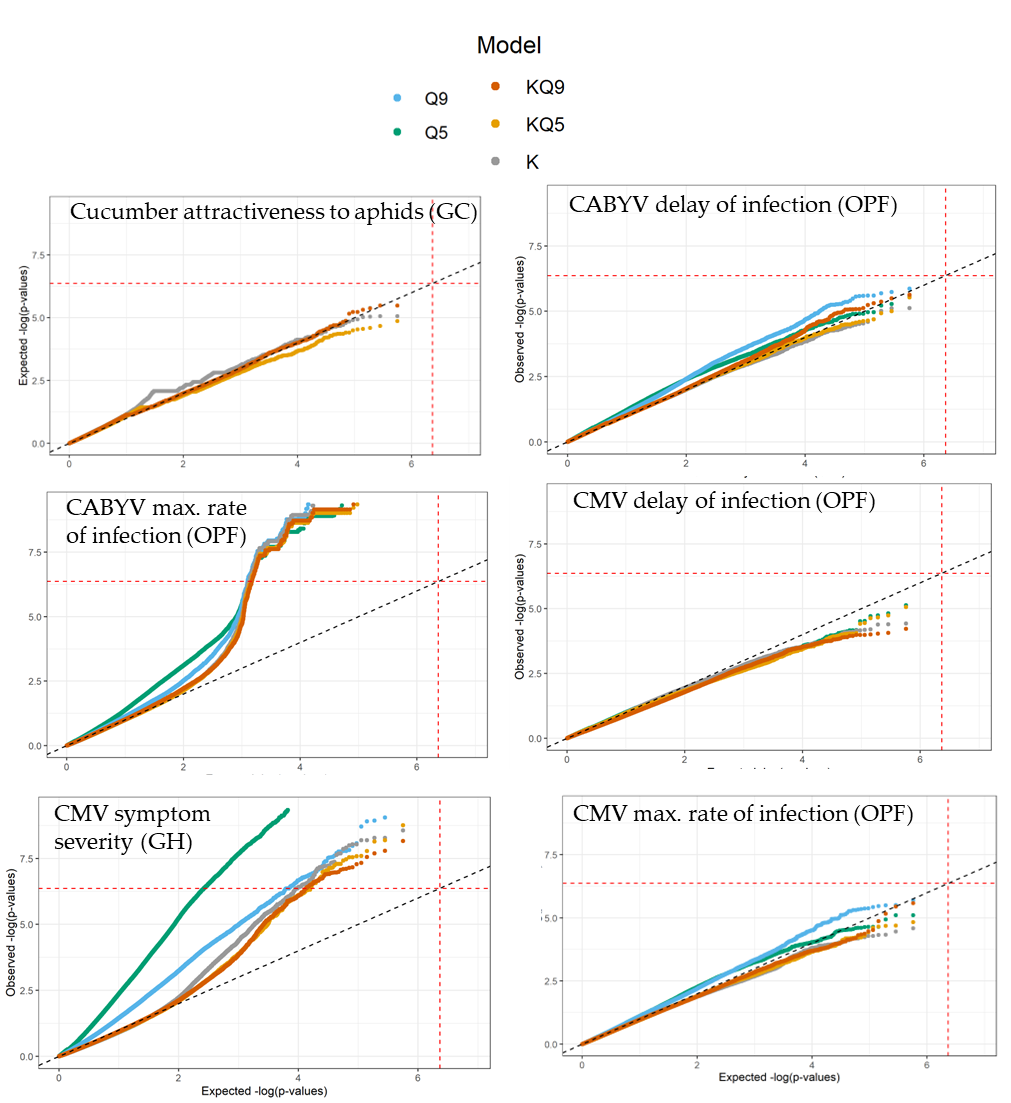


Supplementary figure 7: Qqplots of the Q5, Q9, K, KQ5 and KQ9 models for the nine traits selected.


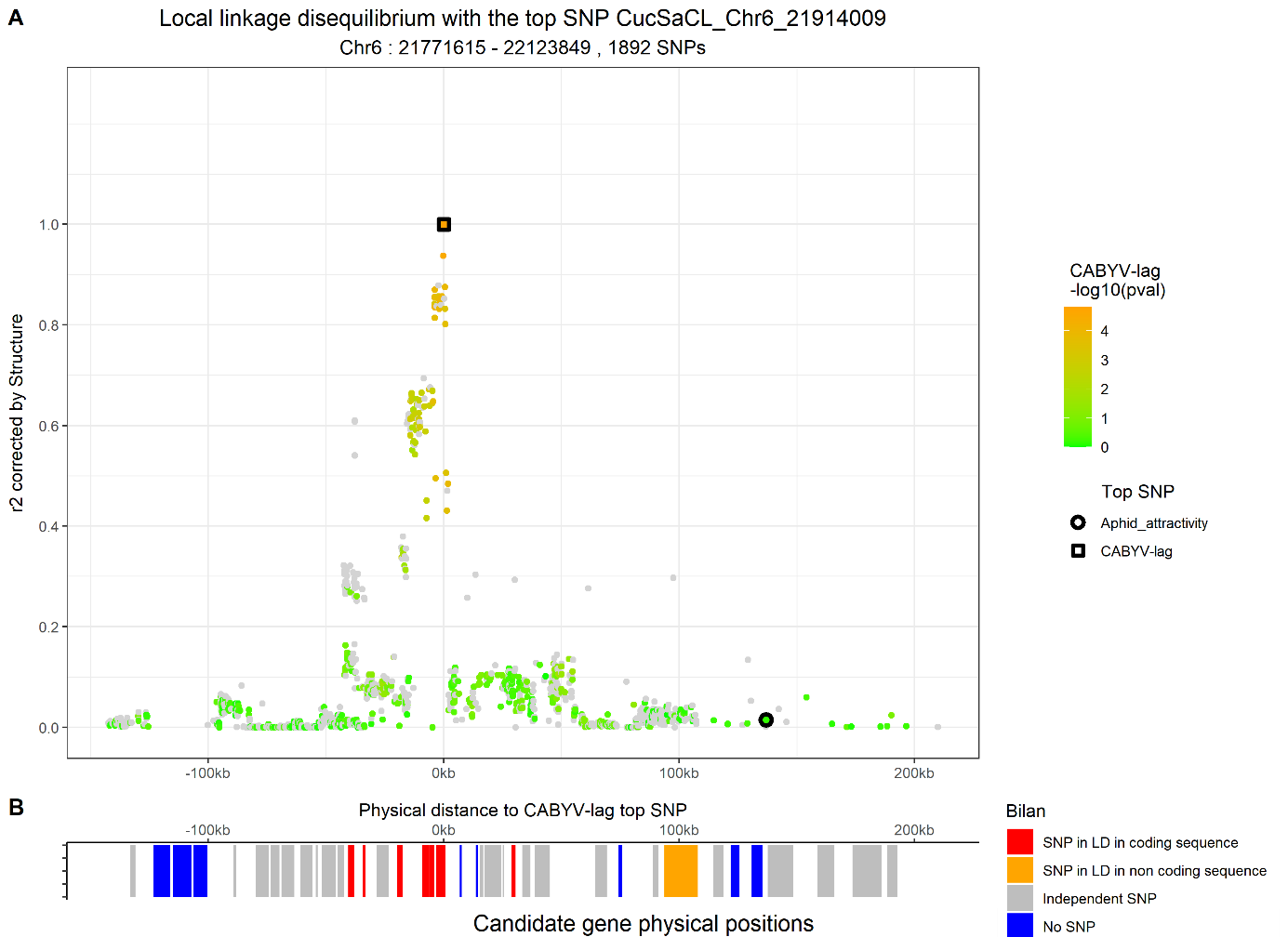


Supplementary figure 8: Local LD study in the cluster of QTLs on chromosome 6 for CABYV delay of infection and aphid attractiveness. A. each point represents a SNP, on the y-axis the r2 with the top SNP of the QTL represented by a dark point. Grey points represent SNP with at least one missing data that were not studied by GWAS. The redder is the point, the more significant is the association. B. Classification of genes located in the QTL interval according to the presence of SNP in LD with the top SNP in coding or non-coding sequence


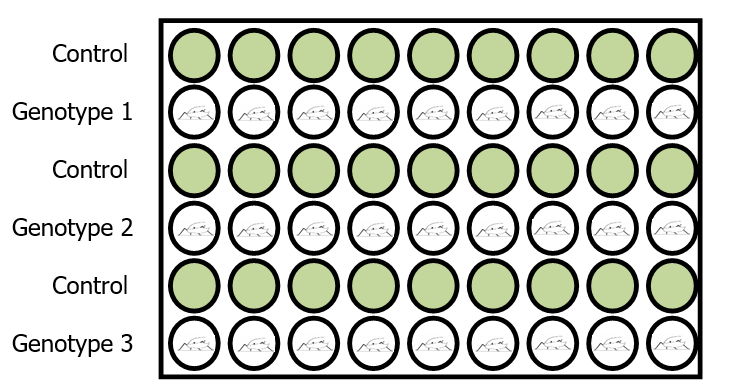


Supplementary figure 9: Leaf disk disposition in the cucumber attractivity to aphid bioassay. Three genotypes from the diversity panel and the repulsive variety ‘Cornell Chinese Long’ are tested at once. One aphid is placed on each leaf disk of genotypes from the diversity panel.
